# Supplementary material for: Anti-Fouling and Anti-Biofilm Performance of Self-Polishing Waterborne Polyurethane with Gemini Quaternary Ammonium Salts
Source: Polymers (Basel). 2023 Jan 7;15(2):317. doi: 10.3390/polym15020317 (PMC9865321; doi:10.3390/polym15020317)
Supplement: Supplementary file 1 [file polymers-15-00317-s001.zip › polymers-2105503-supplementary.pdf]

Supplementary Materials: Anti-Fouling and Anti-Biofilm Performance of Self-Polishing Waterborne Polyurethane with Gemini Quaternary Ammonium Salts

Yi Zhang [1,2], Tao Ge [1], Yifan Li [2], Jinlin Lu [1], Hao Du [1], Ling Yan [3], Hong Tan [2], Jiehua Li [2,*] and Yansheng Yin [1,*]

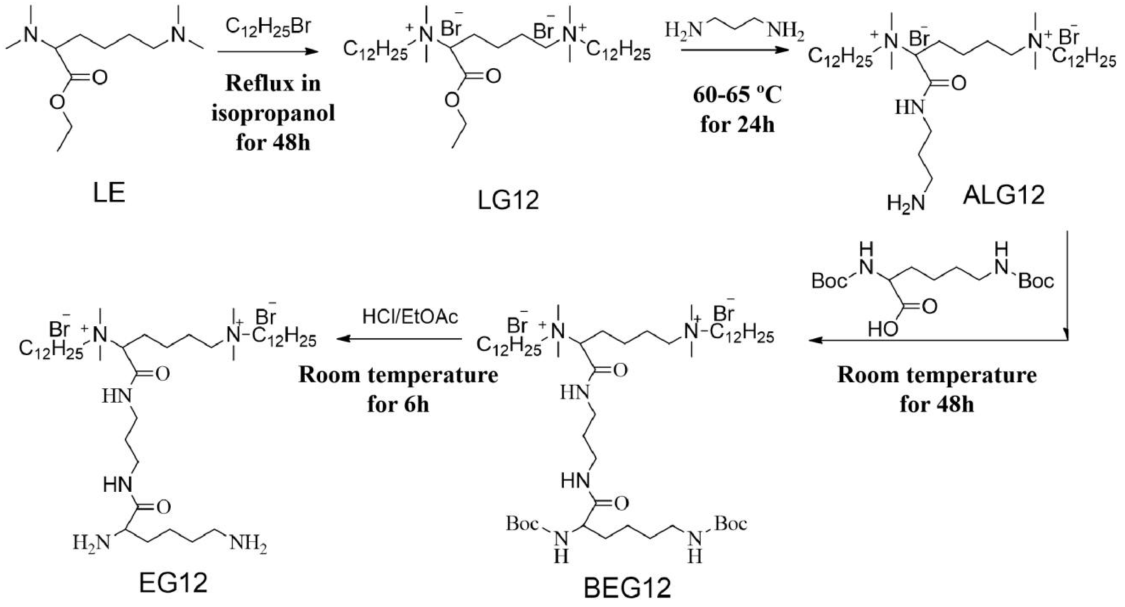

Scheme S1. The synthesis route of gemini quaternary ammonium (GQAS) chain extender (EG12).

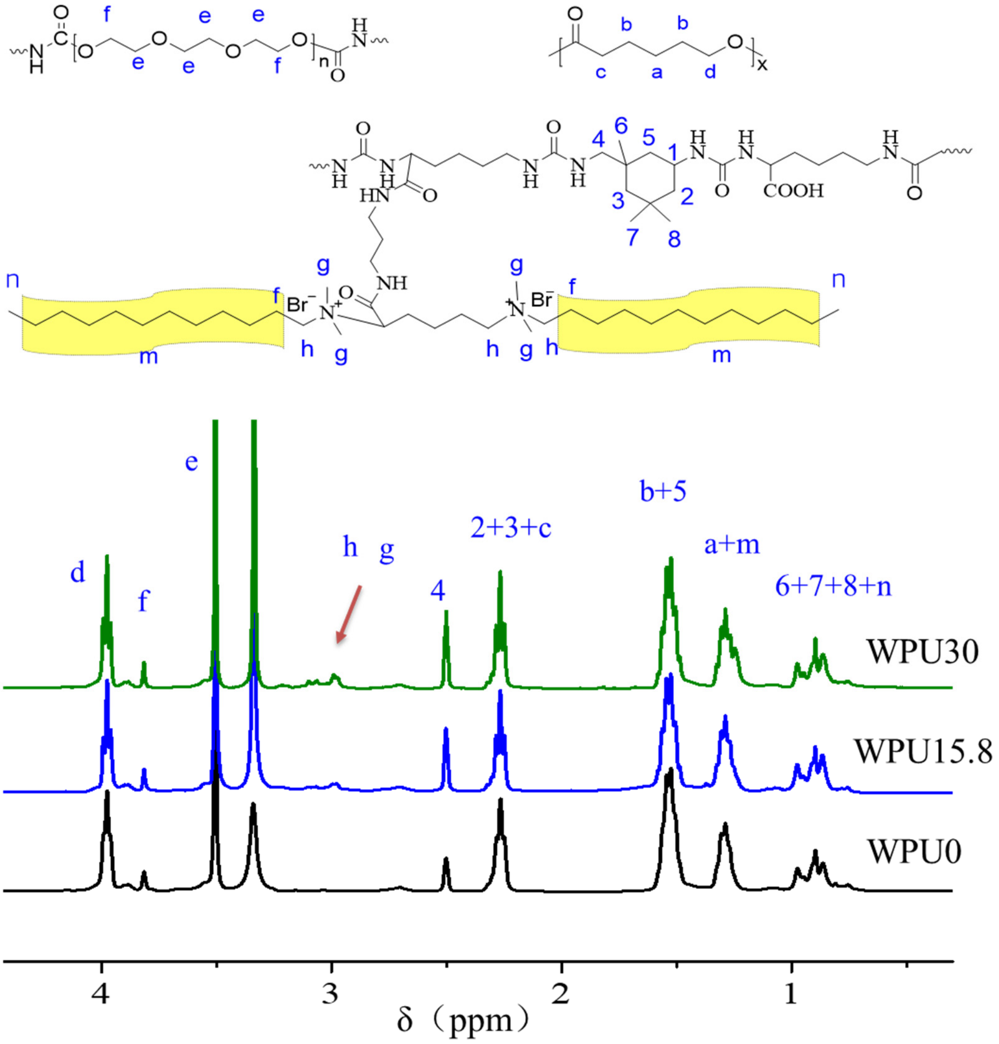

Figure S1. The structures and ^{1}H NMR spectra of WPUn recorded in DMSO-δ6.
